# The Lingering Effects of an Artificial Blind Spot

**DOI:** 10.1371/journal.pone.0000256

**Published:** 2007-02-28

**Authors:** Michael J. Morgan, William McEwan, Joshua Solomon

**Affiliations:** Henry Wellcome Vision Laboratories, Department of Optometry and Visual Science, City University, London, United Kingdom; Istituto di Neurofisiologia, Italy

## Abstract

**Background:**

When steady fixation is maintained on the centre of a large patch of texture, holes in the periphery of the texture rapidly fade from awareness, producing artificial scotomata (i.e., invisible areas of reduced vision, like the natural ‘blind spot’). There has been considerable controversy about whether this apparent ‘filling in’ depends on a low-level or high-level visual process. Evidence for an active process is that when the texture around the scotomata is suddenly removed, phantasms of the texture appear *within* the previous scotomata.

**Methodology:**

To see if these phantasms were equivalent to real low-level signals, we measured contrast discrimination for real dynamic texture patches presented on top of the phantasms.

**Principal Findings:**

Phantasm intensity varied with adapting contrast. Contrast discrimination depended on both (real) pedestal contrast and phantasm intensity, in a manner indicative of a common sensory threshold. The phantasms showed inter-ocular transfer, proving that their effects are cortical rather than retinal.

**Conclusions:**

We show that this effect is consistent with a tonic spreading of the adapting texture into the scotomata, coupled with some overall loss of sensitivity. Our results support the view that ‘filling in’ happens at an early stage of visual processing, quite possibly in primary visual cortex (V1).

## Introduction

Peripheral visual stimuli appear to fade into the background with steady fixation [1], [2]. This ‘Troxler fading’ could be due to fatigue in the mechanisms for detecting the stimuli, or to an active ‘filling-in’ process, mediated by lateral connections [3]. A particularly dramatic kind of Troxler fading is seen with the rapid disappearance of ‘holes’ in flickering noise in peripheral vision. When the adapting texture is suddenly replaced by a blank field, phantasms appear where the holes had been [4]. The perceptual similarity between these phantasms and the adapting texture lends credence to the theory of filling-in. The suggestion is that at least some of the mechanisms responsible for the perception of flickering noise must become activated without direct visual stimulation. However, the observations carried out on these phenomena have so far been only qualitative and subjective. We wondered whether the phantasms would have any psychophysical consequences. In particular, we wondered whether filling-in occurred sufficiently early in the visual pathway for phantasms to affect signal processing in the same way as real signals.

**Figure 1:**
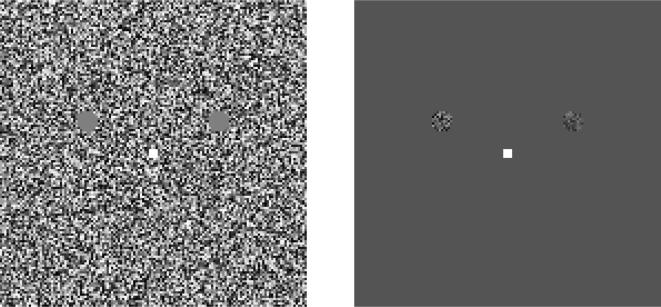
Stimuli for adaptation (a) and test (b) phases of the main experiment. When the observer indicates that the two blank areas have faded from view, the adapting stimulus is replaced for 100 ms with the test stimulus. The observer's task is to decide which of the two test patches (left or right) has the higher contrast. When one of the tests has zero contrast, the task is one of simple detection. Otherwise, it is contrast discrimination.

Contrast discrimination with low-contrast stimuli bears the hallmark of a sensory threshold, below which nothing can be seen. Consider what happens when an observer has to report whether the left or right stimulus has greater contrast (see Fig. 1). As the smaller (i.e. pedestal) contrast increases from zero, it remains subthreshold and thus invisible, but the additional contrast required to exceed the threshold will get lower and lower. Consequently, the difference between the two contrasts required for, say, 82% accuracy will also decrease. (NB: these latter contrasts are known as performance thresholds, not to be confused with sensory thresholds!) An analogy may be drawn with a system involving two teapots. When they are both empty, a drop of water put into one of them produces no discernable difference in output at the spout; but when they are both full, an added drop to one of them can be detected.

Small pedestals thus facilitate discrimination performance. However, above a certain level, pedestals start to decrease sensitivity: an effect called ‘masking’, which is assumed to result from a compressive nonlinearity in contrast signal processing. Thus, performance thresholds first fall, and then rise, creating a ‘dipper’ shaped function as pedestal contrast increases from zero [5], [6].

We wondered whether the phantasms that follow artificial scotomata could act like real pedestals and help to push otherwise invisible stimuli over the sensory threshold. To find out, we measured contrast-discrimination functions using patches of dynamic visual noise that were superimposed on the phantasms. If the phantasms actually exceed sensory threshold, then the real pedestals should cause no further facilitation, and the function mapping pedestal contrast to performance threshold should lose its “dipper” shape.

## Results and Discussion

The stimulus arrangement is shown in Fig. 1. We verified earlier qualitative observations [4] without difficulty. After approximately 5 s of steadily fixating a maximum-contrast noise field, the two target patches disappeared, so that the noise field appeared to be uniform. When the noise was subsequently switched off and replaced by a mean-luminance field, the target areas appeared to be occupied by smudgy, flickering noise not unlike the original noise in appearance. To measure the effect of these phantasms, we switched off the adapting noise for 100 msec, and presented real signals on top of the phantasms. After each 100 msec test the adapting noise came on again immediately, and stayed on until the observer pressed a button to indicate that the scotoma had faded into invisibility.

**Figure 2:**
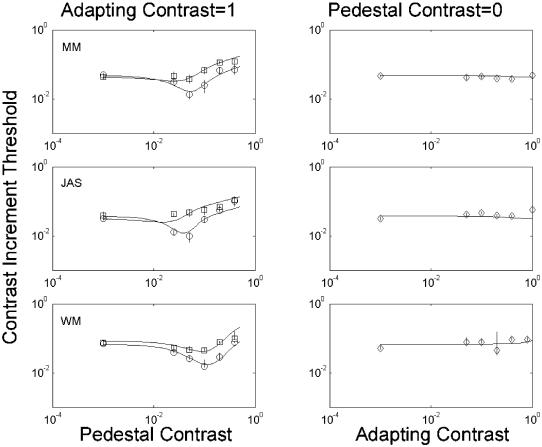
Results of main experiment. Each row shows results for a different observer. The data points are the 82%-correct thresholds derived from psychometric functions, with error bars representing 95% confidence intervals derived from a bootstrap procedure. In the left-hand panels, circles show contrast-discrimination functions in the baseline condition without adaptation. The leftmost point is the detection threshold, at zero pedestal contrast. Boxes show thresholds obtained with a 100%-contrast adapting stimulus. The right-hand panels show how simple detection thresholds (vertical axis) vary as a function of adapting contrast (horizontal axis). The solid curves show best fits of a 6-parameter model, in which sensitivity and ‘filling in’ were allowed to vary with adapting contrast.

The left column in Fig. 2 shows how adaptation affected contrast discrimination. Circles reflect control performance without an adapting field, and rectangles reflect performance after adapting to a maximum-contrast noise field. (In this and subsequent figures, a value of 10° indicates maximum contrast.) The leftmost points in each panel reflect detection (i.e. the actual pedestal contrast was zero). As predicted, adaptation greatly reduced or even abolished the ‘dip’. We attribute this to the phantasms acting like real signals to bring the total response to signal+phantasm above threshold, and this is the model we fit to the data in the continuous curves of Fig. 2. However, if the phantasms had merely helped otherwise-invisible stimuli over the sensory threshold, then detection should have been easier. That is, the leftmost point on the graphs should have fallen. This did not happen. Why?

We suggest that adaptation has *two* effects. One is to produce a phantasm that exceeds the sensory threshold; the other is to reduce overall sensitivity. Sensitivity reduction is a well-documented result of various types of adaptation (e.g. [7]) and there is a solid physiological basis for it: the responses of neurones selective for specific stimuli become reduced following prolonged exposure to those stimuli (e.g. [8], [9]). Nonetheless, the situation here is noteworthy because our results suggest a reduction in sensitivity to contrast increments following adaptation to contrast decrements (‘holes’).

Adaptation to maximum-contrast noise fields produces very strong phantasms. When these phantasms affected contrast discrimination, the effect was always an impairment; that is, adaptation never lowered an observer's performance threshold. We wondered whether this result would generalise to weaker phantasms, elicited by less intense adapting stimuli. To find out, we varied the contrast of the adapting stimulus and measured the performance threshold for detecting a patch of texture on the resultant phantasms (right column in Fig. 2). Again we found that adaptation did not lower performance threshold, except possibly for two of the points on the graph of MM's results. This improvement was very small, but significant because we collected a large amount of data. (Note the very small confidence intervals.) We infer that sensitivity reduction usually outweighs any benefit observers might otherwise enjoy from subthreshold summation.

The continuous curves in Fig. 2 show the best fits of a model, in which both the effective strength of the phantasms as a pedestal and the overall sensitivity were permitted to vary. (Details appear in the Modelling section of Materials and Methods.)

**Figure 3:**
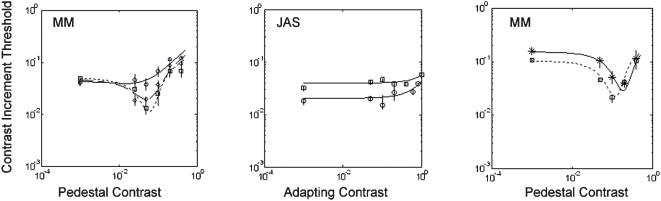
Left panel: The dipper function for MJM at three different levels of adapting contrast (diamonds, squares and circles for adapting contrast 0, 0.3 and 1.0 respectively). Horizontal axis: pedestal contrast; vertical axis, performance threshold. Middle panel: effects of different levels of adapting contrast (horizontal axis) on thresholds at two levels of pedestal contrast (squares and circles). Right panel: Effects of pedestal contrast on contrast discrimination for a 2 cpd/4 Hz drifting Gabor pattern, in the presence (circles) and absence (boxes) of superimposed dynamic noise masks (as in Fig 1b).

To investigate the effects of adaptation contrast on the whole contrast-discrimination function, we performed an experiment on one observer (MM) with adapting contrasts of 0, 0.3 and 1.0. Results are shown in the left panel of Fig. 3. At the medium adapting contrast, the dip was flattened by not abolished. All the data were well fit by the same model as in Fig 2, where phantasms were equivalent to a real pedestal, and adaptation caused an overall reduction in sensitivity. For the leftmost points on the graph (zero real pedestal) sensitivity was higher in the presence of phantasms, but the effect was small and not statistically significant. We also measured (for JAS, middle panel) the effects of adapting contrast at two different pedestal values (0 and 0.02), Facilitation by the pedestal was lost at high adapting contrasts only, in agreement with the results in Fig. 2.

Abolition of the dip, with no effect on detection is, to the best of our knowledge, unprecedented. We wondered whether the phantasms might be reducing uncertainty by informing the observer of the potential target locations. To investigate this, we measured the effects of imitation phantasms (i.e. small, brief patches of real dynamic noise) on contrast discrimination for spatio-temporally co-extensive drifting gratings (Fig. 3 right panel). Any reduction in intrinsic uncertainty regarding the spatiotemporal positions of low-contrast targets should be accompanied by a reduction in facilitation [10]. However, consistent with the results of previous work using larger noise fields [11], facilitation was not lost: the noise merely moved the dip upwards and rightwards. We conclude that the effects of the phantasms should not be attributed to spatiotemporal uncertainty reduction.

**Figure 4:**
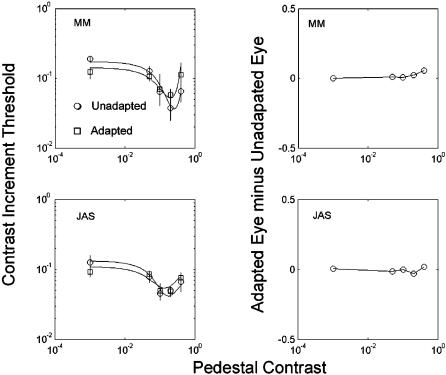
Results of a dichoptic experiment in which the adapting stimulus was in one eye while the other eye saw a blank field. Thresholds as a function of pedestal contrast were then obtained either in the adapted eye or the non-adapted eye. The left-hand panels show the mean (across eyes) for the adapted and non-adapted conditions. The continuous lines are maximum-likelihood fits to these data using the model described in Methods. The effect of adaptation is similar to that of a lower contrast binocular adapter (Fig. 3, top panel). Note that adaptation improves detection performance (leftmost point) in both observers. The right-hand panel shows the difference in threshold between the adapted and the non-adapted eye, in the adapted condition. There is evidence for slightly greater masking at high pedestal levels in the adapted eye, but not at low and intermediate pedestal contrasts.

On the basis of phenomenological reports, Ramachandran & Gregory [4] concluded that the lingering effects of artificial scotomata were cortical in origin. Reich et al [12] came to a similar conclusion using a dichoptic stimulus. We sought further evidence. In an experiment in most respects like the main experiment (Fig. 2) two observers (MM, JAS) performed an experiment where the pedestals and target were either in the same or the different eye from the adapting scotomata (dichoptic presentation, achieved with ferromagnetic shutters exposing the two eyes alternately at a 120 Hz frame rate, in synchrony with the visual display). If the phantasms were affecting signal processing only at the retinal level, there should have been no effect of left-eye phantasms on right-eye signals. Instead, the results (Fig 4) indicate very little difference between adapted and non-adapted eyes. That “very little difference” appears only with high-contrast pedestals. The data suggest slightly less facilitation when these pedestals appear in the adapted eye. To determine whether these differences between the adapted and non-adapted eye were significant, we re-fit our model, using either the same or different parameter values for the two eyes. We found that doubling the number of free parameters did not produce a significant improvement (p>0.1; df = 4). This finding is consistent with there being no significant difference between the adapted and nonadapted eye, and thus with complete interocular transfer. We conclude that the phantasms affected signal processing in the cortex, the first place at which signals from the two eyes converge on common neurones [13].

At first, sight, the results of the last experiment may appear to contradict the results of Experiment 1, in that the dip was still present after adaptation, rather than being abolished. However, it can be argued that the effective adapting contrast at a binocular site was only 0.5 in the dichoptic case, because of the presence of the stimulus in only one eye (the other eye viewed a mean-luminance, uniform field), and we have already shown (Fig. 3 left panel) that the dip is still present after a binocular adapting contrast of 0.3. There is a remaining discrepancy in that the phantasms in the dichoptic case did improve performance in the absence of a real pedestal (leftmost points in each panel). The improvement was significant for MM (Chi-squared = 20.41; df = 2; p<0.001), but not for JAS (Chi-squared = 3.64; df = 2; 0.1>p>0.05). However, the difference between the experiments was quantitative rather than qualitative, in that MM did show an improvement in sensitivity in Experiment 1 (Fig. 2, right hand column) and Experiment 2 (Fig. 3, top panel), albeit smaller than in the dichoptic experiment. We do not have an account of this quantitative difference.

### Conclusions

We conclude that the visual phantasm is accompanied by changes in visual performance like those wrought by real retinal signals, and by adaptation to real signals. The loss of sensitivity is most likely a result of adapting the mechanisms for detecting a texture boundary, and has been previously reported in cortical neurones adapted to moving stimuli [9] This same loss of sensitivity could be responsible, in part, for disappearance of the ‘hole’ during adaptation. More interesting is the effect of adaptation in subthreshold summation, an effect never previously reported, and consistent with ‘filling-in’. We take ‘filling in’ to refer to the lateral propagation of a signal into neurones not receiving a direct input from the stimulus. The site of the sensory threshold must be in these neurones themselves, rather than earlier or later in the pathway, to account for our data. Our cross-eye adaptation experiment rules out an earlier site than V1, and a later site for the threshold would not be consistent with at least one neuroimaging study [14]. We therefore suggest that ‘filling in’ starts in V1 itself. However, this clearly does not mean that the subjective experience of filling in takes place in V1 alone. A previous electrophysiological study [15] has found evidence of a process called ‘climbing activity’, which may well be related to filling-in, in areas V2 and V3 of macaque, but there is scant evidence for such a process in V1. This does not rule out V1 as the site of the threshold, but it may mean that filling-in is a complicated process with several aspects. Indeed, in this paper we have identified at least two consequences of adapting to an artifical scotoma: an overall loss of sensitivity in the adapted area, and abolition of the ‘hard’ threshold for contrast discrimination.

## Materials and Methods

Stimuli were computed with MATLAB and displayed by a Cambridge Research System (CRS) VSG 2/3 graphics card on a Mitsubishi DiamondPro monitor with a refresh rate of 120 Hz. In the final experiment, dichoptic separation was achieved by CRS ferromagnetic goggles, which alternately occluded each eye during the monitor's vertical blanking interval.

The observers were the three authors. MM and JAS are experienced psychophysical observers. WM was an undergraduate student carrying out the experiment as part of a BSc degree in Biology at University College London.

During each trial of the main experiment, an observer fixated on a white spot in the centre of a 23×23 deg square filled with dynamic visual noise. The luminance of each pixel (or group of 4×4 pixels for MM and JAS) was randomly selected from a uniform distribution centred on 33.5 cd/m^2^, 25 times per second. Two blank areas of mean luminance were centred 2.5 deg above and 5 deg to the left and right of fixation. These areas were disk shaped with diameter 1.5 deg (see Fig. 1). The observer was instructed to press a button when the blank areas faded from view. Button presses were followed immediately by 100-ms exposures of similarly distributed dynamic noise, confined to the same disk-shaped areas. The observer's task was to decide which of these two areas had noise with greater contrast.

Trials were blocked by the contrast of the adapting noise. An adaptive staircase[16] determined the increment contrast most likely to produce a response with 82% accuracy. For MM and JAS, the contrast used on each trial was randomly selected from a 4-dB interval centred on this threshold estimate, allowing for a better estimate of psychometric slope.

### Modelling

Our model was based on a standard 4-parameter model of the dipper function [17], with the addition of a further parameter *s*, to represent an added contrast signal due to ‘filling in’. The model computes a neural response *R*, to a stimulus contrast *c*.
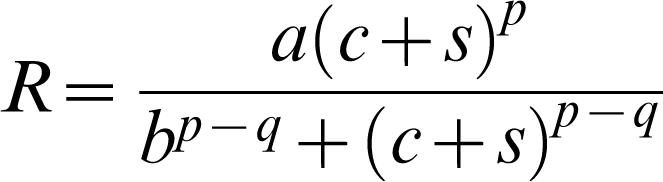
1


The five parameters in this formula are: *a*, which determines overall sensitivity; the exponent *p* which gives rise to facilitation with low-contrast pedestals; the exponent *q*, which gives rise to masking with high-contrast pedestals; *b*, which determines the pedestal contrast at which facilitation gives way to masking; and *s*, explained above, On each trial the probability of a correct response *p_i_*, was then computed from the normal integral:
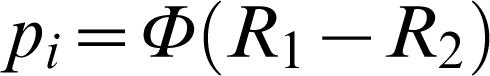
2Where *R*_1_ is the response to the larger signal and *R*_2_ is the response to the smaller signal.

The likelihood of obtaining the observed response probability was computed by:
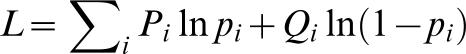
3where *P_i_* and *Q_i_* denote the number of correct and incorrect responses, respectively. Finally, the values of the parameters maximizing the summed log-likelihoods were found by gradient descent, using the MATLAB^@^ ‘fminsearch’ function.
